# A comparison of euphemistic strategies applied by MBC4 and Netflix to two Arabic subtitled versions of the US sitcom *how I Met Your Mother*

**DOI:** 10.1016/j.heliyon.2021.e06262

**Published:** 2021-02-15

**Authors:** Hanan Al-Jabri, Areej Allawzi, Abdallah Abushmaes

**Affiliations:** a Department of English Language and Literature, University of Jordan, Amman, Jordan; b Linguistic Research Manager, Mawdoo3, Amman, Jordan

**Keywords:** Audiovisual translation, Euphemism, Politeness, Satellite TV channels, Video streaming services

## Abstract

This paper explores the different sets of strategies applied by two different media outlets in subtitling taboo terms from English into Arabic. The study sets out to examine if different subtitling policy is adopted in each media outlet to deal with taboo words based on the social and religious limitations expected to be found in the Arab society. The assumption the study makes is that Arabic satellite TV channels, unlike video streaming services, receive interference from religious, political, and social authorities to maintain a “clean” content and censored language of the shows they air. To achieve this goal, the study compares two different Arabic subtitles of the taboo terms used in the American sitcom *How I Met Your Mother.* The first translation was produced by the Arabic TV channel MBC4 and the second by the subscription-based video streaming service Netflix. The study draws on the euphemistic strategies suggested by Williams (1975) and Warren (1992) and further developed by Al-Adwan (2015) to analyze the Arabic subtitles produced by each media outlet. The findings of the study indicate that the Arabic subtitles produced by the satellite Arabic TV channel reflect a higher level of politeness where euphemism, as a politeness strategy, was clearly applied by the subtitler to avoid rude and embarrassing terms.

## 1 Introduction

The evolvement of television industry is considered a main factor which has contributed to the globalization of the world into a small village. Almost every household nowadays has at least one television that communicates information and entertainment and gathers family members to watch their favorite shows. This growth has increased the demand on importing foreign shows to appeal to the viewers, which in turn increased the demand on translating these shows into the viewers' first language. Therefore, a new mode of translation, namely audiovisual translation (AVT), has been introduced into television to overcome the language barriers and facilitate the travel of information and entertainment across geographical borders. González (2009: 13) defined AVT as “a specialized branch of translation which deals with the transfer of multimodal and multimedial texts into another language and/or culture”. The business of AVT witnessed yet another huge leap upon the vast growth of subscription-based video streaming services, such as Netflix which is available in almost all countries in the world which, therefore, requires fast translation of its shows into multiple languages.

Although AVT helped spread entertainment among different countries with different languages, cultural barriers proved stronger than the geographical ones; a barrier which has always been an obstacle when it comes to translation in all its different modes. This cultural barrier seems to aggravate the problem when the two languages/cultures involved are distant, such as English and Arabic. Therefore, the subtitler is expected to work out technical, linguistic, and cultural obstacles. One of these cultural problems is taboo words.

It is no secret that many factors come into play when Arabic TV channels choose to air a foreign series. Political and religious authorities have a great influence on the content of these shows and what should and should not be aired. Probably this influence is due to the religious nature of the Arab society which does not accept the public discussion of certain taboo areas including sex, drugs, and gay relationships, among others (Al-Yasin and Rabab'ah, 2019). The language of foreign shows normally goes through a filtering process to purify it from offensive, embarrassing, or shocking terms. Video streaming services, particularly Netflix, on the other hand, is rather an independent streaming service. The shows it streams cover a wide range of topics which are considered taboos in many cultures including the Arab one and, therefore, does not seem to have similar limitations. Moreover, TV viewers have little or no control over what they watch on TV channels, which are accessible by all people with different age groups and backgrounds. Video streaming service subscribers, on the other hand, choose to subscribe to these services and, therefore, choose to view shows that might be obscene, violent, or offensive.

If subscription-based services, particularly Netflix, seem to enjoy more liberty in the content it streams worldwide, it is probably safe to assume that same level of liberty is expected to be practiced in its subtitling policy into different languages including Arabic. To test this assumption, the current study aims to compare the euphemisation strategies used in two Arabic subtitled versions of the American sitcom *How I Met Your Mother* produced by the Arabic satellite TV channel MBC4 and the video streaming service Netflix. The study argues that two different sets of translation strategies were applied in the two Arabic versions of the American sitcom based on the different level of liberty each media outlet enjoys. The comparative analysis between the two Arabic versions of the American show will be conducted in three main categories of taboo areas including sex-related terms, same sex-related terms, and private body organs.

## 2 Review of related literature

As discussed earlier in the introduction, AVT has made a huge contribution to the TV industry. With the translation of foreign shows into different languages, information and entertainment crossed language barriers. According to Khuddro (2018: 10), AVT covers two main modes; audio translation including dubbing or voice-over, and visual translation including subtitles using television, cinema, and other devices such as computers and mobile phones. Dubbing is an oral translation activity which depends on the use of the acoustic channel in screen translation (Baker and Hochel, 2001) while subtitling is the process of transferring a source language (SL) audiovisual media to a target language (TL) synchronized with the original verbal message (Gottlieb, 2004). According to Gambier (2013), selecting either mode was normally determined by various economic, ideological and pragmatic factors. Translation business seems to prefer subtitling for financial reasons as it costs less than dubbing. It also provides more credibility and joy for the audience who experience original sounds.

The job of the subtitler is considered by many scholars as more complicated than that of the translator; it does not only require a high level of linguistic and cultural knowledge between the two languages, but also the possession of a wide range of other technical skills (Hussain and Khuddro, 2016; Khuddro, 2018). According to Kruger (2008: 82):

Subtitling requires all the skills that other modes require in terms of text analysis,

subject expertise, language, awareness of context, quality control and so forth, but it

also requires that the subtitler to be able to apply these skills within very rigid

constraints of time and space, while adhering to specific conventions of quantity and

form.

AVT, as a subject area, according to several scholars, is relatively new (Al-Adwan, 2015). De Meo (2010: 19), for example, points out that the area of AVT “has only recently fully been recognized in translation study research as previously it was merely considered as an inferior form of adaptation”. While a growing interest in AVT research can be seen in many European countries, little has been done in the Arab world, where to the best of the researcher's knowledge, few researchers have explored some cultural and linguistic issues in Arabic subtitling (see Gamal, 2008; 2009; Al-Adwan, 2009; 2015; Thawabteh, 2011; Khuddro, 2018). Also, according to the researcher's knowledge, very few studies have been done to investigate the different translation strategies applied by satellite TV channels in comparison to those applied by video streaming services when dealing with taboo terms in the Arab culture. Therefore, this paper aims to fill this gap and contribute to this academic field in both video streaming services and satellite TV channels.

Language and culture are inseparable in any translation activity. Therefore, not only should the translator/subtitler have knowledge of the linguistic systems of both languages but also knowledge of both cultures to produce the most accurate and meaningful message in the TL. However, cultural items are not always straightforward in the process of translation. Culture is perceived differently and, therefore, problems arise. The cultural problem which this paper aims to tackle is the rendition of taboo words. According to Allan (2001: 148), “tabooed words are those considered offensive, shocking, or indecent when used in certain contexts”. However, the problem with taboos is that people's beliefs and values differ from one culture to another and therefore their perception of taboos differs. According to Elmgrab (2020: 23), the nature of taboo words is vague which intensifies the problem of translating them between different languages. Some expressions, Elmgrab (2020: 23) adds, “are considered taboo and may elicit embarrassment or offence to certain people whereas they are used naturally by other people.” The problem with translating taboo words does not lie in finding equivalents in the TL because, in some cases, one may find direct equivalents; the problem perhaps lies in the impact of using these direct equivalents on the target audience, which can be offensive and unacceptable even if accurate. Taboo words are considered taboo for a reason; they offend the audience and are culturally insensitive.

These cultural differences in perceiving taboo words probably apply best to the English and Arabic cultures. While the West culture seems more accepting of using taboo words in movies and series, and does not mind the daring sexual scenes, it is very unlikely to accept this practice in the Arabic media. A good example of this taboo intolerance in the Arabic media is the Jordanian series “Jinn” which was produced by Netflix in 2019. The series, which was aired in the Jordanian dialect, contained several taboo words, particularly very blunt insults and swear words, in addition to some intimate scenes between teenage school students, such as hugging and kissing. Although these insults are widely used among the youth in schools and almost everyone knows what they mean, the Jordanian community was furious at the series for “publicly” using them to the extent that they demanded the government to interfere and ban Netflix in Jordan. If the degree of acceptance of these taboo words differs according to the social and cultural norms and expectations, translating them is, therefore, subject to almost similar practice.

Therefore, it can be concluded that the nature of taboo words differs from one culture to another and many factors come into play when determining what can be said and what cannot be said in a certain culture. However, almost every society has a set of taboos which act as a broad umbrella under which different words or topics are considered prohibited in public. In this study, the classification of taboo areas is inspired by several previous studies which discussed taboo language in the Arab culture (see Farghal, 1995; Al-Adwan, 2009; 2015; Thawabteh, 2017; Al-Adwan and Yahiaoui, 2018). They mention that taboos are names of prohibited things and acts that are associated mainly with customs and political authority and which cannot be discussed in public. These taboos cover subjects such as religion, sex (including sexual behavior, sex organs, gay relationships), bodily functions (such as urinating and defecating), unpleasant matters (such as death, misfortunes, and suicide), profanities, and alcoholism.

According to Toury (1995), taboo words require the Arab translators to use one of two options: foreignization which means finding an adequate equivalent for the taboo word that acts with the norms of SL, or domestication which is finding a euphemistic equivalent that conforms to the norms of TL. Euphemism, according to Abrantes (2005: 86), is defined as ‘‘a word or a phrase used in a specific linguistic and extra-linguistic context to soften or conceal something unpleasant.’’ Similarly, Cruse (2006: 57) defines euphemism as “an expression that refers to something that people hesitate to mention lest it cause offence, but which lessens the offensiveness by referring indirectly in some way.” Communicators in all cultures, although differ in their perception of taboos, employ euphemistic expressions in specific contexts. Therefore, euphemisms, as Brown and Levinson (1987: 2016) argue, “are a universal feature of language usage.’’

As discussed above, therefore, euphemism is a politeness strategy used by communicators to tone down the effect of inappropriate words or taboos in a particular culture or a particular context. Al-Adwan (2015: 8) maintains that euphemism has always been applied by Arab audiovisual translators to avoid committing face threatening acts that may distort the public image of Arab viewers. Therefore, when a potential threat is evoked by a taboo word, euphemism is resorted to by the subtitler to avoid any unpleasant encounter.

### 2.1 Euphemism strategies

Many researchers and scholars examined euphemisms and euphemisation strategies (see Enright, 1985; Farghal, 1995; Huang, 2005; Gamal, 2008; Al-Adwan, 2009; 2015; Thawabteh, 2017). However, the current study will rely mainly on the euphemisation model strategies suggested by Williams (1975) and Warren (1992) and further developed by Al-Adwan (2015) to conduct the comparative analysis between the two Arabic versions of the American sitcom. These strategies include:

1 **Widening**: This strategy involves substituting the offensive word with a more general term that is less offensive. An example of this strategy, as mentioned by Linfoot-Ham (2005: 232), is using the word *satisfaction* to refer to *orgasm.* In this case, orgasm is a more specific word that is replaced by *satisfaction*, a more general term that can be achieved through various ways among which is orgasm.
2 **Implication:** According to Al-Adwan (2015: 11), implication involves two propositions, where the second is usually a logical consequence of the first. Normally, the euphemisms generated through this process has a conventional meaning, and, at the same time, implies a novel interpretation. A good example offered by Warren (1992) is the euphemism *loose*, which conventionally means *unattached*, and which generates the potential novel interpretation (sexually easy/available).
3 **Metonyms:** Although this process is similar to widening as both result in general substitutions, metonyms, as explained by Al-Adwan (2015: 13), are metonymically related to the items they substitute. In other words, both words hold a whole-part relationship between their established and novel referents.
4 **Omission:** As the term suggests, this strategy occurs when subtitlers/translators decide to remove a word that is deemed to be seriously offensive or face threatening.

## 3 Data and methodology

The selected American series *How I Met Your Mother* is a comedy show consisting of nine seasons that ran from 2005 to 2014. The data used in this study consist of the first season which comprises 22 episodes. The show depicts the life of a group of friends, namely Ted, Marshall, Barney, Lili, and Robin who live in Manhattan, New York. The events of the series are narrated as a flashback by the main character Ted Mosby after getting old in the year 2030 recounting to his son and daughter the events happened between 2005 to 2013 that led him to meet their mother.

The researcher chose this particular sitcom for the purposes of this study for a variety of reasons. First of all, the characters in the show are all in their late twenties who deal with daily topics including dating, marriage, professional career, socializing and so forth. This wide range of topics and situations yield diverse use of verbal exchanges including the use of taboos or culturally unacceptable terms making the show a rich research material. Second, the data are available and accessible online; the first Arabic subtitled version of the series is available on Netflix while the other one is available on MBC4. Finally, the availability of the subtitled series on two different media platforms is in fact a valuable opportunity for conducting contrastive studies that trace many verbal and cultural behaviors between the West and the East.

As pointed out earlier, the current study aims to compare two Arabic subtitled versions of the American sitcom *How I Met Your Mother* to explore the translation strategies applied in each one to deal with taboo terms used in the American sitcom. It aims at revealing how the subtitling practice differs between a video streaming service and a satellite TV channel assuming that the TV channel version is expected to be more polite.

The researcher used the following procedure to collect and analyze the data:

1. Watching the original American series and manually collecting all taboo words which fall into the selected categories outlined in the introduction. Al-Adwan (2009: 123) points out that the taboo area of sex includes a wide range of topics among which are romantic and sexual relationships, body parts (especially organs related to sex) and sexual orientation. The current study will deal only with these taboo areas.
2. Classifying the taboo words detected in the data into the three categories mentioned earlier: sex-related terms (words/phrases referring to sexual relationships), same sex-related terms, and private body organs.
3. Exploring the Arabic subtitles produced by Netflix and MBC4 to examine how the selected taboo words were subtitled into Arabic by both media outlets.
4. Identifying the translation strategies used in the two Arabic versions to render the taboo words from English into Arabic.
5. Discussing the differences identified in the Arabic subtitles produced by Netflix and MBC4 and highlighting the pattern of translation detected in each media outlet.

## 4 Findings and discussion

As explained earlier, the current study focuses on three categories of taboos which are likely to cause problems in the process of translation. Each section will discuss one of these categories providing relevant examples found in the corpus. The categories are sex-related terms, same sex-related terms, and private body organs.

### 4.1 Sex-related terms

Sex, although a popular subject in human communications, is considered a source of embarrassment and discomfort in many cultures, especially if discussed publicly. However, the degree of threat caused by this subject differs from one society to another (Khalaf and Gagnon, 2006). In the Arab culture, sex is threatening to the face of most Arab interlocutors probably due to the Islamic background of this society. According to Al-Yasin and Rabab'ah (2019: 3), “the Arab culture, in general, is well known to be a conservative one especially with the impact of religion on society. Therefore, it is unusual to hear a plain-spoken taboo word in the Arab media”. Concepts such as dating and having sexual relationships before marriage are prohibited and considered sins in Islam. The sitcom, therefore, is expected to cause a threat to the face of Arab viewers as it openly discusses these subjects. References to sex found in the first season probably surpass references to all other taboo subjects. This section will shed light on all sex-related terms used in the first season. The most popular references referring to the act of sexual activity detected in the corpus include *have sex with*, *hook up with*, *sleep with*, *get laid*, and *nail*. See Table 1 below.

**Table 1:** Season 1. References to sex.

No.	English Text	MBC4	Netflix
1.	Ted to Marshal: Yes, perfect. And then you’re engaged. You pop the champagne and you drink a toast and you **have sex** on the kitchen floor. Please don’t **have sex** on the kitchen floor.	تيد: نعم ممتاز وبعدها ستصبح رجلاً مخطوباً وستفتح زجاجة شمبانيا وتشرب نخباً و**تمارسان علاقة** على أرضية المطبخ. لا **تمارسا العلاقة** على أرضية المطبخ. *Yes, perfect. And then you’re an engaged man and you’ll pop a champagne bottle and you drink a toast and you****have a relationship****on the kitchen floor. Don’t****have a relationship****on the kitchen floor.*	تيد: نعم ممتاز وبعدها ستصبح رجلاً مخطوباً وستفتح زجاجة شمبانيا وتشرب نخباً و**تمارسان الجنس** على أرضية المطبخ. لا **تمارسا الجنس** على أرضية المطبخ. *Yes, perfect. And then you’re an engaged man and you’ll pop a champagne bottle and you drink a toast and you****have sex****on the kitchen floor. Don’t****have sex****on the kitchen floor*.
2.	Robin’s co-worker to her: we should **have sex**. I’m just saying we should **have sex**. **Having sex** is fun.	يجب أن **نقيم علاقة**. أنا قلت فقط يجب أن **نقيم علاقة. إقامة علاقة** ممتعة. we should have **a relationship**. I’m just saying we should **have a relationship**. **Having a fun relationship.**	يجب أن **نمارس الجنس**. أنا قلت فقط يجب أن **نمارس الجنس. ممارسة الجنس** ممتعة. Robin’s co-worker to her: we should **have sex**. I just said we should **have sex**. **Having sex** is fun.
3.	Robin’s co-worker to her: Bummer. I was hoping to finally **have sex with you** this weekend, Scherbatsky.	يا لسوء الحظ. كنت آمل أنني **سأقيم علاقة معك** أخيراً في نهاية هذا الأسبوع "شيرباتسكي". BT: What a bad luck. I was hoping **I would have a relationship with you** finally at the end of this week Scherbatsky.	يا لسوء الحظ. كنت آمل أنني **سأضاجعك** أخيراً في نهاية هذا الأسبوع "شيرباتسكي". BT: What a bad luck. I was hoping **I would lie with you** finally at the end of this week Scherbatsky.
4.	Barney’s ex to him: We **had sex** twice in your car and then you dumped me.	**أقمنا علاقة** مرتين في سيارتك وبعد ذلك تركتني. Barney’s ex to him: **We had a relationship** twice in your car and then you left me.	**مارسنا الجنس** مرتين في سيارتك وبعد ذلك تركتني. Barney’s ex to him: **We had sex** twice in your car and then you left me.
5.	Barney’s ex to Ted: You know, the traditional rain dance is a sacred prayer to nature.I don't think the great spirit looks too kindly on white dudes who co-opt it to **get laid.**	أتعلم رقصة المطر التقليدية هي صلاة مكرسة للطبيعة لا أعتقد أن الروح العظيمة ستستجيب لرجل أبيض يريد المطر **ليقيم علاقة**. You know, the traditional rain dance is a prayer dedicated to nature. I don't think the great spirit will respond to a white dude who wants to **have a relationship.**	أتعلم رقصة المطر التقليدية هي صلاة مكرسة للطبيعة لا أعتقد أن الروح العظيمة ستستجيب لرجل أبيض يريد المطر **ليمارس الجنس**. You know, the traditional rain dance is a prayer dedicated to nature. I don't think the great spirit will respond to a white dude who wants to **have sex**.
6.	Barney to Ted: Think about it, this is perfect. A-- it will make robin insanely jealous. B-- you get to **have sex with her**. And c-- maybe by getting to know Mary, you'll come to see that courtesans are people, too. And d-- **"b"** all night long.	فكر بهذا إنه مثالي. أولا سيجعل روبن تغار بجنون. ثانياً **ستقيم علاقة معها** وثالثا ربما بمعرفتك لماري سترى أن هؤلاء العشيقات بشر أيضاً. ورابعا هو ثانيا **ستقيم علاقة معك** طول الليل. Think about it. This is perfect. First it will make Robin insanely jealous. Second, you get to **have a relationship with her**. And third, maybe by getting to know Mary, you'll come to see that these mistresses are people, too. And fourth is second **she will have a relationship with you** all night long.	فكر بهذا إنه مثالي. أولا سيجعل روبن تغار بجنون. ثانياً **ستمارس الجنس** معها وثالثا ربما بمعرفتك لماري سترى أن هؤلاء العشيقات بشر أيضاً. ورابعا هو ثانيا **ستنام معك** طوال الليل. Think about it. This is perfect. First it will make Robin insanely jealous. Second, you get to **have sex with her**. And third, maybe by getting to know Mary, you'll come to see that these mistresses are people, too. And fourth is second **she will sleep with you** all night long.
7.	Ted to Robin: Are you gonna **hook up with him**?	هل **ستخرجين** معه؟ Are you gonna **go out with him?**	هل **ستمارسين الجنس** معه؟ Are you gonna **have sex with him**?
8.	Robin to Marshall: He lied and told me he broke up with his girlfriend to try to **hook up with me**.	كذب علي وقال إنه انفصل عن حبيبته ليحاول **إقامة علاقة معي**. He lied and told me he broke up with his girlfriend to try to **have a relationship with me**.	كذب علي وقال إنه انفصل عن حبيبته ليحاول **معاشرتي**. He lied and told me he broke up with his girlfriend to try to **have sex with me**.
9.	Barney to Ted: I mean, seriously—Claudia and Stuart? I mean, **I’ve hooked up with the odd lass** who was beneath my level of attractiveness, but, you know, I was drunk. There’s no way Claudia has been drunk for three years.	أعني جدياً (كلاوديا) و(ستيوارت)؟ **أقمت علاقة مع نساء** كن أقل من مستوى جاذبيتي لكنني كنت ثملاً. يستحيل أن تكون كلاوديا ثملة ل3 سنوات. I mean, seriously—Claudia and Stuart? **I’ve had a relationship with women** who were beneath my level of attractiveness, but I was drunk. There’s no way Claudia has been drunk for three years.	أعني جدياً (كلاوديا) و(ستيوارت)؟ أعني **ضاجعت نساء** كن أقل من مستوى جاذبيتي لكن كما تعلمان كنت ثملاً. يستحيل أن تكون كلاوديا ثملة ل3 سنوات. I mean, seriously—Claudia and Stuart? **I’ve had lied with women** who were beneath my level of attractiveness, but I was drunk. There’s no way Claudia has been drunk for three years.
10.	Barney to Ted: You lied and said you were broken up with Victoria before you actually were so you could try to **nail** robin.	كذبت وقلت إنك انفصلت عن فكتوريا قبل أن يحدث ذلك في الواقع كي تتمكن من **إقامة علاقة** مع روبن. You lied and said you broken up with Victoria before it actually happened so you could **have a relationship with** Robin.	كذبت وقلت إنك انفصلت عن فكتوريا قبل أن يحدث ذلك في الواقع كي تتمكن من **معاشرة** روبن. You lied and said you broken up with Victoria before it actually happened so you could **have sex with** Robin.
11.	Barney: Come on, Ted, do it. This is one of those things you have to do before you turn 30. Ted: **Sleep with a prostitute**? Barney: No, **lose your virginity**.	بارني:بربك يال تيد افعلها. هذه أحد الأشياء التي يجب عليك فعلها قبل أن تصل إلى سن ال 30 تيد: **النوم مع بنت هوى**؟ بارني: لا **فقدان عذريتك**. ـــــــــــــــــــــــــــــــــــــــــــــــــــــــــــــــ Barney: Come on, Ted, do it. This is one of those things you have to do before you reach the age 30. Ted: **Sleep with a prostitute**? Barney: No, **lose your virginity**.	بارني: بربك يال تيد افعلها. هذه أحد الأشياء التي يجب عليك فعلها قبل أن تصل إلى سن ال 30 تيد: **إقامة علاقة مع منحلة**؟ بارني: لا **فقدان البراءة**. ـــــــــــــــــــــــــــــــــــــــــــــــــــــــــــــــ Barney: Come on, Ted, do it. This is one of those things you have to do before you reach the age 30. Ted: **have a relationship with a degenerate (woman)**? Barney: No, **lose your innocence.**

Phrases in bold represent examples of sex-related terms in the English version and their Arabic counterparts in the two Arabic versions.

The English phrases *have sex/hook up/sleep with/nail*/*get laid*, although differ in register, are direct references to the act of having sex which trigger a sense of embarrassment and shame, especially for the Arab viewers. Looking at the renditions adopted by the subtitler of MBC4, we notice that every occurrence of any of these phrases is substituted with the more general expression يقيم علاقة ‘have a relationship’ opting, thus, for the strategy of widening. The phrase يقيم علاقة ‘have a relationship’ in Arabic is a broad phrase which refers to romantic relationships between men and women that may or may not involve sex. In the Arab world nowadays, romantic relationships before marriage are not uncommon; pre-marriage sexual relationships are. Therefore, this phrase can be used to refer to either type. In these examples, however, and within the context of these episodes which depict the Western lifestyle with which most Arab people are familiar, it is probably clear that the type of relationship the characters were having are ones involving sex. Moreover, many Arab viewers, even if not well-educated, are familiar with the English word *sex* which, when heard, can contribute to their understanding of the actual meaning of the general term يقيم علاقة ‘have a relationship’. Therefore, the Arab viewers are expected to understand what this phrase means in context.

In example 7, however, the widening strategy applied by MBC4 subtitler seems to affect the intended meaning. In this example, Ted was asking Robin if she was going to have sex with her co-worker. His question conveys jealousy and shock because he loves Robin and wishes she would love him back. The term ستخرجين معه ‘are you going out with him’ was used by MBC4 subtitler to replace ‘hook up with him’ probably to avoid using face threating phrases. Unlike the phrase يقيم علاقة ‘have a relationship’ used normally by MBC4 subtitler, ستخرجين معه ‘going out with him’ connotes the type of relationships where sex is unlikely to be involved, and, therefore, communicates a toned-down meaning.

The subtitler of Netflix, on the other hand, opted mainly for the literal equivalent يمارس الجنس ‘have sex’ to replace many of these phrases. The Arabic phrase, although involves the word جنس ‘sex’, is formal and neutral. In some examples (3/6/8/9), however, the subtitler resorted to phrases which reflect a lower register than the original. For instance, in example 3, the subtitler replaced the neutral English phrase *have sex* with the less formal phrase سأضاجعك ‘I will lie with you’. Similarly, ستنام معك ‘she will sleep with you’ was used in example 6 to replace *have sex*. However, the chosen Arabic phrase seems to fit the overall ‘vulgar’ context where Barney was convincing Ted to have sex with a prostitute.

Other sex-related terms detected in the data and included in this table are the words *prostitute* and *lose your virginity* outlined in example 11. Here Barney is trying to convince Ted to have sex with a prostitute while Ted is rejecting Barney's attempt. Both terms are embarrassing and a source of threat to the face of the Arab viewers. The first term is used to describe a person who engages in a sexual relationship in exchange for money while the latter refers to having sexual intercourse for the first time. Expectedly, the subtitler of Netflix resorts to literal translation using the terms بنت هوى and فقدان عذريتك respectively while MBC4 subtitler opts for the strategy of widening substituting the former with the general term منحلة ‘degenerate’ and the latter with ‘lose innocence’. Linfoot-Ham (2005) explains that euphemisms generated through the strategy of widening are highly context dependant; they need to be particularized in a given context in order to provide interlocutors with logical connotations. For example, منحلة ‘degenerate’ can be used to describe someone who does all types of immoral things including drinking, dressing or acting inappropriately, engaging in sexual intercourse, etc. Nevertheless, Arab viewers can successfully infer that the intended meaning is someone who ‘engages in sexual intercourse’. Similarly, فقدان البراءة ‘lose your innocence’, can be decoded as ‘having sex for the first time’.

As the above examples show, the choices of the MBC4 subtitler reflect deference to the face of the target viewers. Widening is the dominant euphemisation strategy applied by the subtitler to play down the intensity of the blunt phrases referring to sex in the corpus. Although the meaning was generally preserved in these renditions, the level of informality and humor was not. Also, it seems that aiming to censor the taboo content resulted sometimes in awkward structures (see example 2). Netflix subtitler, on the other hand, opted for literal translations. Some of these literal phrases, however, reflect a lower register than the original which can be even more shocking for many Arab viewers.

The following section highlights the second category of taboos found in the corpus.

### 4.2 Same sex-related terms

Same sex relationships, or gay relationships, are another taboo area in the Arab world not only because they are sexual but also because they are religiously prohibited. Although same sex relationships exist in the Arab society, they are viewed as a psychological disorder rather than a personal choice. Thawabteh (2017) maintains that films imported in the Arab market are normally subjected to institutionalized censorship and manipulation for a wide range of sensitive issues including gay relationships.

Table 2 below demonstrates examples of same sex-related terms found in the corpus along with their two Arabic versions.

**Table 2:** Season 1. Same sex-related terms.

No.	English	Netflix	MBC4
1.	Lili's ex-boyfriend: You're, you're breaking up with me? Lili: There's still so much I want to do. I want to travel, live overseas as an artist, maybe have a lesbian relationship.	- هل تنفصلين عني؟ -أمامي الكثير لفعله. أريد السفر والعيش خارجاً كفنانة وربما أدخل في علاقة مثلية. -You're breaking up with me? - There's still so much I want to do. I want to travel, live overseas as an artist and maybe have a same sex relationship.	-هل تنفصلين عني؟ -أمامي الكثير لفعله. أريد السفر والعيش خارجاً كفنانة. -You're breaking up with me? - There's still so much I want to do. I want to travel and live overseas as an artist.
2.	Lili to Robin: I didn't have any of the experiences I set out to. The travel, the bohemian art life, my big lesbian experience. I didn't do any of it. Robin: Lily, you're marrying your best friend in the world. I mean, isn't that worth all the other experiences combined? And look, you can still travel, I mean, you can still paint. And as far as your lesbian experience... (Robin comes close and kisses Lili on her lips)	- لم أخض أي تجربة من التجارب التي خططت لها. السفر، الحياة الفنية البوهيمية وتجربة العلاقة المثلية. لم أفعل شيئاً من ذلك. -ليلي أنت تتزوجين من أفضل صديق لك في العالم. ألا يساوي ذلك كافة التجارب مجتمعة؟ -بلى -وما زال بإمكانك السفر والرسم وبالنسبة إلى علاقتك المثلية... (روبن تقترب وتقبل ليلي على شفتيها) -I didn't have any of the experiences I set out to. The travel, the bohemian art life, my big same sex relationship experience. I didn't do any of it. - Lily, you're marrying your best friend in the world. I mean, isn't that worth all the other experiences combined? you can still travel, you can still paint. And as far as your same sex relationship experience... (Robin comes close and kisses Lili on her lips)	لم أخض أي تجربة من التجارب التي خططت لها. السفر، الحياة الفنية البوهيمية. لم أفعل شيئاً من ذلك. ليلي أنت تتزوجين من أفضل صديق لك في العالم. ألا يساوي ذلك كافة التجارب مجتمعة؟ بلى وما زال بإمكانك السفر والرسم. -I didn't have any of the experiences I set out to. The travel, the bohemian art life. I didn't do any of it. - Lily, you're marrying your best friend in the world. I mean, isn't that worth all the other experiences combined? you can still travel, you can still paint.

The two examples outlined in the table were detected in episode 20 of the first season of the series. Lilli and Robin went to a prom to check the performance of the band Lili was planning to hire for her wedding. The prom seems to bring back memories of when she broke with her high school boyfriend because she had many dreams she wanted to achieve among which was getting involved in a lesbian relationship. Looking at the translations of this taboo term in both examples, we notice that Netflix subtitler opted for the general equivalent علاقة مثلية ‘same sex relationship’ which includes a sexual relationship between two men or two women; in this example, it is very clear it refers to the latter. In formal contexts, علاقة مثلية ‘same sex relationship’ is normally used to refer to all types of gay relationships while there are a few other informal or less preferred terms such as علاقة سحاقية *lesbian relationship* referring particularly to sexual relationships between women. The subtitler of MBC4, however, omitted the phrase altogether. The kiss scene between Robin and Lili was also omitted on MBC4 while it was retained on Netflix.

Similarly, the scene where Barney kissed Marshall on the lips, episode *Pilot*, was retained on Netflix but removed on MBC4. Furthermore, a complete episode, namely episode 10 season 2, was not aired on MBC4 because it involved a gay wedding party between James, Barney's brother, and his fiancé, Tom, who were also planning to adopt a baby. It is worth mentioning, however, that intimate scenes of kissing between male and female characters were not censored on MBC4 which may lead to the conclusion that same sex topics pose a much serious threat to the face of the Arab viewers than regular sex topics. Therefore, probably using the extreme measure of omission is a clear indication of how serious this topic is.

The following section will discuss the last category of taboos which is private body organs.

### 4.3 Private body organs

Private body organs, particularly those associated with sex or bodily functions, are considered taboos by many cultures including the Arab one (Farghal, 1995). Therefore, mentioning these organs explicitly in public is expected to evoke a sense of embarrassment or discomfort for most Arab viewers of the sitcom. Table 3 below outlines the examples of taboo terms in this category along with their Arabic translations produced by both media outlets.

**Table 3:** Season 1; private body organs.

No.	English	Netflix	MBC4
1.	Robin: Wow. That is one bad-ass blue French horn. Ted: Yeah. Sort of looks like a Smurf **penis**.	روبن: ذلك البوق الفرنسي الأزرق رائع. تيد: نعم. يشبه **قضيب** أحد السنافر نوعاً ما. -Wow. That is a wonder blue French horn. - Yeah sort of looks like a Smurf **penis**.	روبن: ذلك البوق الفرنسي الأزرق رائع. تيد: نعم. يشبه أحد السنافر نوعاً ما. -Wow. That is a wonder blue French horn. - Yeah sort of looks like a Smurf.
2.	*Narrator*: son, a piece of advice. When you go on a first date, you really don't want to say Smurf **penis**.	تيد: يا بني، سأعطيك نصيحة صغيرة. عندما تكون في أول موعد غرامي، فالأفضل ألا تتحدث عن “**قضيب** السنافر”. Son, I'll give you a piece of advice. When you go on a first date, it is better not to talk about Smurf **penis**.	تيد: يا بني، سأعطيك نصيحة صغيرة. عندما تكون في أول موعد غرامي، فالأفضل ألا تتحدث عن السنافر. Son, I'll give you a piece of advice. When you go on a first date, it is better not to talk about the Smurfs.
3.	*Robin*: All right, what does VIP stand for in your little universe? *Barney*: well, I know that **“P” is Penis.**	روبين: حسناً، ماذا يعني مصطلح الشخصيات المهمة في عالمك الصغير؟ بارني: أعرف أن “بي” تعني **عضو ذكري**. All right, what does VIP stand for in your little universe? -Well, I know that **“P” is masculine organ.**	روبين: حسناً، ماذا يعني مصطلح الشخصيات المهمة في عالمك الصغير؟ بارني: أعرف أن “ميم” تعني **المنطقة الحساسة**. All right, what does VIP stand for in your little universe? Well, I know that **“S” means sensitive area.**
4.	*Robin*: I just wrapped up a live newscast by honking my own **boobs**.	روبين: لقد أفسدت للتو بثاً حي ومباشر بإمساكي **لثديي**. I just ruined a live newscast by holding my own **breasts**.	روبين: لقد أفسدت للتو بثاً حي ومباشر بإمساكي **لصدري**. I just ruined a live newscast by holding my own **chest**.
5.	*Narrator*: and so, Barney was right. The night was legendary. It would come to be known as the time Lily kicked Korean Elvis in **the nards**.	وهكذا كان بارني محقاً. كانت الليلة أسطورية. سيعرف هذا الوقت باسم الوقت الذي ركلت فيه (ليلي) **خصيتا** (كورين إلفيس). and so, Barney was right. The night was legendary. It would come to be known as the time Lily kicked Koreen Elvis in **the nards**.	وهكذا كان بارني محقاً. كانت الليلة أسطورية. سيعرف هذا الوقت بأنه الوقت الذي ركلت فيه (ليلي) **المنطقة الخاصة** ل (إلفيس) الكوري. and so, Barney was right. The night was legendary. It would come to be known as the time Lily kicked the **private area** of Korean Elvis.
6.	Lili: What? Ted: lily you're being a wee bit intense about this band thing. Lili: Intense? I have a wedding to plan in nine weeks for 200 people. Even if a dinosaur should poke his head out of my butt and consume this coffee table, I need you to roll with it, okay?	ماذا؟ ليلي إنك تبالغين في حدتك بخصوص الفرقة. حدة؟ لدي زفاف لأنظمه خلال 9 أسابيع لدعوة 200 شخص. حتى لو أخرج دايناصور رأسه من مؤخرتي وشرب كل هذه القهوة على الطاولة أحتاجك أن تتماشى مع الأمر. حسنا؟ What? - Lily you're exaggerating how intense you are about this band thing. Lili: Intense? I have a wedding to plan in nine weeks for 200 people. Even if a dinosaur should poke his head out of my **butt** and consume this coffee in the table, I need you to roll with it, okay?	ماذا؟ ليلي إنك تبالغين في حدتك بخصوص الفرقة. حدة؟ لدي زفاف لأنظمه خلال 9 أسابيع لدعوة 200 شخص. حتى لو أخرج دايناصور رأسه من جسدي وشرب كل هذه القهوة على الطاولة أحتاجد أن تتماشى مع الأمر. حسنا؟ What? - Lily you're exaggerating how intense you are about this band thing. Lili: Intense? I have a wedding to plan in nine weeks for 200 people. Even if a dinosaur should poke his head out of **my body** and consume this coffee in the table, I need you to roll with it, okay?

Phrases in bold represent examples of private body organs and their Arabic counterparts.

Examining the Arabic subtitles used to render terms of private body organs produced by Netflix and MBC4, one notices a significant difference. The main euphemisation strategies detected in the selected examples are widening, omission, and metonymy. In the first two examples, Ted and Robin go out on their first date to a restaurant where they saw a weird shaped French horn. Ted joked about the way it looked saying it resembles a smurf penis which caused an awkward situation for Robin.

The subtitler of MBC4 omitted the taboo word *penis* probably to mitigate any discomfort evoked by this explicit use of the embarrassing term while the subtitler of Netflix retained the term using قضيب which matches the English one in register. As far as the meaning is concerned, the omission of the taboo term in these examples seems to be confusing to the viewers who would not understand the reason behind the awkward situation caused by Ted's comment about the smurfs and, therefore, the level of humor is mitigated in the scene.

In the third example, we notice that *penis* is rendered as المنطقة الحساسة ‘sensitive area’ by MBC4 subtitler and as عضو ذكري ‘masculine organ’ by Netflix subtitler. The former choice reflects the strategy of widening; المنطقة الحساسة ‘sensitive area’ in Arabic is a general term which may refer to any male or female sexual organ. In this example, and since Barney was talking about himself, the viewers can decode what it means successfully and, therefore, the meaning was delivered. Although the choice of Netflix is more explicit than MBC4, it is more formal than the original. However, the structure is rather awkward. The subtitler failed to use the accurate Arabic letter standing for the first letter in the phrase عضو ذكري ‘masculine organ’ which is ‘ع’ rendering it as بي ‘b’ (which is a literal rendition of the original) unlike the accurate letter used by MBC4 subtitler. Similar awkward structure is evident in Netflix rendition of *Korean Elvis* where *Korean* is transliterated into كورين ‘Koreen’, which is not comprehensible for the Arab viewers as the nationality but rather as a foreign name.

The same strategy of widening can also be observed in the renditions of MBC4 subtitler in examples 4 and 5 where صدري ‘chest’ and المنطقة الخاصة ‘private area’ replace the original *boobs* and *nards*. Both words used by the subtitler can be decoded by the Arab viewers from the context. Although the subtitler of Netflix opted for literal translation as he/she usually does, the Arabic word ثديي ‘breasts’ reflects a higher register than *boobs*. Arabic has informal words referring to a woman's breasts, but they are rather colloquial and would be awkward if used within a standard language context. This is probably why the subtitler used ثديي ‘breasts’ instead.

The strategy of metonymy, on the other hand, was detected in the last example. The specific part *ass* was replaced with the “whole’, namely Liliy's body. The subtitler manages to save the face of the viewers through this choice, while he/she mitigates the intensity of the scene; Lili was angry and nervous planning her wedding, and using this informal taboo word by her reflects her level of anger which was toned down by the euphemized alternative.

The analysis of this section shows that the strategies used by MBC4 subtitler to translate private body organs were widening, omission, and metonymy. Literal translation, on the other hand, was the main strategy used by Netflix subtitler although some of the words reflect a higher register than the original probably due to the nature of the Arabic language where the informal alternatives do not seem to fit within a standard language context.

## 5 Conclusion

This study investigated the translation strategies used by the subtitlers of MBC4 and Netflix in delivering taboo words found in the first season of the American sitcom *How I Met Your Mother*. The author assumed that the version produced by Netflix would show less euphemisation strategies based on the content normally streamed by the video streaming service. A range of examples extracted from the first season of the series were classified into three categories of taboo areas: sex-related terms, same sex-related terms, private body organs. These topics as explained earlier are considered taboos in the Arab culture and could potentially damage and threaten the face of Arab viewers. The analysis embarked on the euphemisation model developed by Al-Adwan (2015) to account for the strategies applied by both subtitlers to mitigate the face threatening terms found in the corpus.

The findings of the study support the assumption that Netflix Arabic version of the series seems to have undergone little or no censorship; the subtitler opted in most examples for literal equivalents to render the taboo terms found in the corpus although the choices sometimes reflected a different register and a more formal style than the original English words. This stylistic difference can perhaps be attributed to the fact that many informal or vulgar words in Arabic are colloquial that cannot be standardized and, therefore, cannot be used in mass media where standard Arabic is usually used. Although these standard choices are limited to formal contexts and are not normally used in informal daily conversations among Arab speakers, they can still be comprehended and, therefore, can reflect the intended meaning and effect.

On the other hand, the subtitler of MBC4 did not use any literal equivalents for any of the taboo terms. Instead, the subtitle used a set of euphemisation strategies. Widening was the main strategy detected to tackle the face threatening terms in the first category of taboos. All references to sex were replaced by more general terms that can be easily decoded from the context. However, in some situations, the intensity of the scenes and the level of humor were mitigated due to using this strategy. The subtitler did not resort to Arabic formal equivalents either, as the case in Netflix Arabic version, which contributed to mitigating the intended humor effect on the viewers. Omission was the dominant strategy in the second category of same sex-related terms. Lesbian relationships and same sex intimate scenes were all removed from the show affecting the level of humor. The extreme measure of omission seems to reflect the seriousness and sensitivity of this topic in the Arab culture. Finally, widening, metonymy, and omission were used by the subtitler to render private body organs. As explained earlier in the discussion, private body organs are related to bodily functions and sexual activity making them another taboo area in many cultures including the Arab one. These results are consistent with the findings of other research papers conducted by other scholars including Al-Adwan (2015).

Needless to say that the Arab society nowadays is a conservative and religious one which does not welcome using or hearing offensive, shocking, or rude terms related to the three categories outlined in the study. Therefore, it was expected that the satellite TV channel owned by Arab people and operating in the Arab world, where political and religious authorities have a say over its content, would have to resort to euphemisation strategies to mitigate the intensity and offensiveness of the taboo terms used in the foreign series. On the other hand, Netflix does not seem to be under the same restrictions and, therefore, the Arabic subtitles reflect this level of freedom.

## Declarations

### Author contribution statement

H. Al-Jabri: Analyzed and interpreted the data; Contributed reagents, materials, analysis tools or data; Wrote the paper.

A. Allawzi: Conceived and designed the experiments.

A. Abushmaes: Performed the experiments.

### Funding statement

This work was supported by the 10.13039/501100005712University of Jordan.

### Data availability statement

Data included in article/supplementary material/referenced in article.

### Declaration of interests statement

The authors declare no conflict of interest.

### Additional information

No additional information is available for this paper.
